# Symmetric spatiotemporal learning network with sparse meter graph for short-term energy-consumption prediction in manufacturing systems

**DOI:** 10.1016/j.heliyon.2024.e34394

**Published:** 2024-07-10

**Authors:** Jianhua Guo, Mingdong Han, Chunlin Xu, Peng Liang, Shaopeng Liu, Zhenghong Xiao, Guozhi Zhan, Hao Yang

**Affiliations:** a School of Computer Science, Guangdong Polytechnic Normal University, Guangzhou, Guangdong, China; b Computer College, Hangzhou Dianzi University, Hangzhou, Zhejiang, China

**Keywords:** Energy-consumption prediction, Deep learning network, Manufacturing system, Graph convolutional network, Gated linear unit

## Abstract

Short-term energy-consumption prediction is the basis of anomaly detection, real-time scheduling, and energy-saving control in manufacturing systems. Most existing methods focus on single-node energy-consumption prediction and suffer from difficult parameter collection and modelling. Although several methods have been presented for multinode energy-consumption prediction, their prediction performance needs to be improved owing to a lack of appropriate knowledge guidance and learning networks for complex spatiotemporal relationships. This study presents a symmetric spatiotemporal learning network (SSTLN) with a sparse meter graph (SMG) (SSTLN-SMG) that aims to predict multiple nodes based on energy-consumption time series and general process knowledge. The SMG expresses process knowledge by abstracting production nodes, material flows, and energy usage, and provides initial guidance for the SSTLN to extract spatial features. SSTLN, a symmetrical stack of graph convolutional networks (GCN) and gated linear units (GLU), is devised to achieve a trade-off not only between spatial and temporal feature extraction but also between detail capture and noise suppression. Extensive experiments were performed using datasets from an aluminium profile plant. The experimental results demonstrate that the proposed method allows multinode energy-consumption prediction with less prediction error than state-of-the-art methods, methods with deformed meter graphs, and methods with deformed learning networks.

## Highlights

• An effective method for short-term energy-consumption prediction in manufacturing systems was presented.
• A sparse meter graph was devised to guide spatial feature extraction of the energy-consumption time series.
• A symmetric spatiotemporal learning network was devised to extract features from an energy-consumption time series.
• The proposed method is a collaborative multinode energy-consumption prediction method based on a time series.

## 1 Introduction

Global warming and fossil fuel depletion have exerted significant pressure on the manufacturing industry to pursue energy-efficient development [1]. Nowadays, an increasing number of manufacturing systems have implemented the industrial internet, enabling them to collect energy-consumption data at the machine and minute levels, thereby furnishing detailed information for effective energy management. Short-term (minutely or hourly) energy-consumption prediction serves as the foundation for various applications, including anomaly detection, real-time scheduling, and energy-saving control. It has emerged as a prominent research focus in recent times [[2], [3], [4], [5]]. Large-scale manufacturing systems are generally composed of thousands of machines that consume multiple classes of energy, such as power, gas, fuel, steam, and compressed air. Moreover, interface or management limitations generally make it difficult to predict energy-consumption using process details. Therefore, short-term energy-consumption prediction at the machine level faces challenges such as high prediction frequency, a large number of prediction nodes, limited data acquisition, and little prior knowledge. Furthermore, prediction methods must consider the spatiotemporal relationships between production nodes (machines, process units, and workshops), which are complicated by flexible process paths and dynamic production rhythms [2,[6], [7], [8]].

Existing energy-consumption prediction methods for manufacturing systems can be categorised in three ways: output, input, and modelling. Based on the output, they can be categorised into single and multinode methods. In the former method, a prediction model is built only for a single production node (a single machine, process unit, or workshop). In the latter, a prediction model is shared among multiple production nodes. Therefore, multinode methods can deal more effectively with a high prediction frequency and a large number of prediction nodes.

According to the input, existing methods can be categorised into parameter- and sequence-based methods. Parameter-based methods input relevant parameters, such as yields, process states, and weather conditions, and attempt to capture the relationship between the parameters and energy consumption through machine learning or mechanism analysis [3,4,[9], [10], [11]]. However, they generally suffer from limited parameter acquisition in actual manufacturing systems. Sequence-based methods only input historical energy-consumption time series and attempt to capture temporal features from the time series using machine learning. They do not suffer from limitations in data acquisition.

According to the modelling, existing methods can be categorised into physics-based and learning-based methods. Physics-based methods rely on physical principles and detailed parameters of machines [9,[12], [13], [14]], which are difficult to acquire. Learning-based methods rely on machine learning models and energy-consumption relationships, and do not suffer from limited data acquisition and little prior knowledge.

Owing to the aforementioned advantages, the motivation of this study is to present a multinode, sequence-based, and learning-based method for short-term energy-consumption prediction in manufacturing systems. The key task is to construct a learning model to capture the complex spatiotemporal relationships among production nodes.

Classical sequence-learning-based models include the autoregressive integrated moving average (ARIMA) model [15], XGBoost model [9], Kalman filtering model [16], support vector regression (SVR) model [[17], [18], [19]], and back-propagation (BP) neural network model [20]. These models cannot fully capture complex features from big data and generally provide unsatisfactory prediction accuracy. In recent years, recurrent neural networks (RNNs) [21] and their improvements, such as long short-term memory (LSTM) [22] and gated recurrent units (GRU) [23,24], have been widely applied in energy-consumption prediction, such as power consumption prediction in cement raw material grinding systems [25], building energy-consumption prediction [[26], [27], [28]], and regional natural gas consumption prediction [29]. These methods extract the periodic patterns of time series elaborately but ignore the spatial relationships among production nodes.

Multinode energy-consumption prediction in manufacturing systems is a class of multivariate time-series prediction that generally focuses on capturing spatiotemporal relationships among variates. A few methods for multivariate time-series prediction integrate GRU (or LSTM) with a convolutional neural network (CNN) to capture spatiotemporal relationships among variates [[30], [31], [32], [33], [34]]. They attempt to extract the spatial features by performing convolutional kernels, which slide over regularly arranged multivariate time series and compute weighted sums of values to create feature maps. However, CNNs are commonly used for Euclidean data, such as images and regular grids, and cannot work well in the context of multinode energy-consumption prediction owing to irregular node relationships. Other related methods employ attention-based RNNs to capture the spatiotemporal relationships between variates [[35], [36], [37], [38], [39]]. In these methods, an attention mechanism based on an encoder-decoder model is employed to learn the spatial relationship between exogenous and target series. However, the attention mechanism is generally used to extract the correlation between different series and does not work well for solving time-series autoregressive problems.

The graph convolutional network (GCN) is a novel method to capture hidden spatial relationships from multivariate time series. It is specifically designed to handle graph-structured data, which lacks the regularity and translational invariance of Euclidean data. In a graph, each node may have a different number of neighbours, making it challenging to apply CNN operations. Instead, GCNs focus on extracting structural features by defining local receptive fields in a way that accounts for the irregularity of the graph. This can be achieved through spatial methods, which consider the neighbouring nodes and edges of each node, or spectral methods, which operate on the graph's Laplacian matrix to capture global structural information [40]. Many methods combine GCN with RNN, CNN, or other temporal convolution modules to capture spatiotemporal relationships among variates. The construction of a graph is the basic task of graph convolution. Generally, each variate is considered a node, with each node forming the spatial relationship with the corresponding edge. The graphs can be summarised into two categories: (1) pre-constructed graphs and (2) dynamic graphs.

Pre-constructed graphs are generally the inputs of the learning model and are constructed based on prior knowledge. They are mostly found in applications with clear topologies, such as power grid load prediction [2,3] and traffic flow prediction [[41], [42], [43], [44]]. In these methods, pre-constructed graphs express the explicit spatial relationships between variates, time series express the state evolution of the graphs, and they lead to the extraction of implicit spatial and temporal relationships through graph and temporal convolution. Specially, a study fuses a few spatial graphs with different time signals into a spatiotemporal graph, and then captures the complex localized spatiotemporal relationships through node embedding. It achieved good performance in traffic flow prediction [45]. Pre-constructed graphs combine human knowledge and machine learning and can achieve better prediction performance than dynamic graphs in many scenarios. However, existing graph construction methods for energy consumption in manufacturing systems mostly focus on Petri nets, which describe process events such as machine failure, lack of material, and changeovers [[46], [47], [48]]. These methods rely on a great deal of prior knowledge and go beyond resource modelling; therefore, they are not applicable to the problems in this study.

Dynamic graphs are generally the modules of a learning model in which each variate (node) is represented as an embedded vector, and the connections between variates are determined by the distance between the embedded vectors [[49], [50], [51], [52]]. The embedded vectors are randomly initialised [49,50,53], or initialised as linear transformations of the input time windows [51,52], and then optimised based on gradient descent. Therefore, the graph structure is adaptive over the time window and reflects the correlation of the fluctuations between the time series. However, for energy consumption in manufacturing systems, false correlations may be captured in a dynamic graph, which reduces prediction accuracy. For example, two parallel machines may show highly correlated energy-consumption series because they frequently share the same functionality and work rhythm; however, their energy consumptions should be independent of each other because there is no material flow or ownership between them.

After constructing a variate graph, developing a spatiotemporal learning network is another task that includes the temporal convolution module, spatiotemporal module layout, and channel number assignment. The majority of existing methods employ GRU (or LSTM), empirical module layout, and empirical channel number; e.g., “T-S-T” and “S-T-S-T” module layouts are employed in Refs. [2,42], respectively, where T denotes temporal convolution and S denotes spatial convolution. However, empirical structures are only appropriate for specific scenarios. For short-term energy-consumption prediction, a learning network must balance spatiotemporal feature extraction and suppress noise interference.

In summary, to extract spatiotemporal features for short-term energy-consumption prediction in manufacturing systems, CNN-based methods are not well suited for the irregular node relationships, attention-based methods are not well suited for the time-series autoregression, and dynamic graph-based methods may lead into false correlations. Pre-constructed graph-based methods are promising owing to the combination of prior knowledge and machine learning. However, two problems must be solved: an appropriate meter graph (**Q1**) and an appropriate learning network (**Q2**).

This paper presents a symmetric spatiotemporal learning network (SSTLN) with a sparse meter graph (SMG) (SSTLN-SMG), which is a novel multinode-sequence-learning-based method for short-term energy-consumption prediction in manufacturing systems. The main contributions of this study are as follows.

(1) A set of concepts is defined for multinode-sequence-learning-based energy-consumption prediction, and modelling tasks are clarified by associating input, output, and prediction models with machines, materials, and production in manufacturing systems.
(2) A multistep evolving method for SMGs is presented, in which the complex spatial relationships between energy meters are expressed by abstracting the hierarchical structure, material flows, and energy usage. Compared with existing Petri net based methods, the presented method takes resource-centric modelling instead of process-centric modelling, does not rely on process details and expresses explicit meter relationships in an intuitive way. (**Solution to Q1**)
(3) A novel learning model, the SSTLN, is presented to enable accurate multinode energy-consumption prediction, in which the combination of a graph convolutional network (GCN) and gated linear unit (GLU) is employed to extract spatiotemporal features from energy-consumption time series using SMG. Different from existing spatiotemporal learning networks, a symmetrical module layout instead of an empirical layout is employed to trade-off not only between spatial feature extraction and temporal feature extraction but also between detail capture and noise suppression. (**Solution to Q2**)

The remainder of this paper is organised as follows. The problem is defined in Section 2. The construction method for the SMG is presented in Section 3. The SSTLN method is presented in Section 4. Section 5 describes an application case. Interpretations of the experimental results are discussed in Section 6, and the conclusions are presented in Section 7.

## 2 Problem definition

This section is to clarify the **Q1** and **Q2** by formally defining the input and output of the problem, especially the explicit spatial relationships of the input. In simple terms, the inputs and outputs are historical and future energy consumption time series, respectively. To improve the prediction accuracy, the time series need to be connected by production relationships. It is assumed that a manufacturing system is composed of numerous machines connected by material flow. One machine consumes numerous classes of energy, and each class of energy consumption is measured minutely by a smart meter, and each meter maintains an energy-consumption time series. These concepts are defined as follows.

Definition 1 Production Nodes **P**. A production node refers to a hierarchical management unit such as a plant, workshop, line, machine group, or individual machine. The machine is the production node at the lowest level. **P =** {*p*_1_,*p*_2_, …,*p*_*L*_}, **P** is the set of production nodes in the manufacturing system, and L is the size of **P**.

Definition 2 Edges between Production Nodes **Ep. Ep =** {*ep*_*ij*_|*p*_*i*_,*p*_*j*_ ∈ **P**, i≠j },where ep_ij_ is the edge between p_i_ and p_j_ and only exists if there is direct material flow between p_i_ and p_j_.

Definition 3 Production Graph **Gp**. An unweighted graph **Gp** = (**P**, **Ep**) was used to describe the topological structure of the material flow.

Definition 4 Meter Nodes **M**. An energy meter measured the energy consumption of a production node. **M =** {*m*_1_,*m*_2_, …,*m*_*N*_}, **M** is the set of energy meters in the manufacturing system, and N (≥*L*) is the size of **M**. There is a one-to-many relationship between production nodes and meters.

Definition 5 Edges between Meter Nodes **Em. Em** = {*em*_*ij*_| *m*_*i*_,*m*_*j*_ ∈ **M**, *m*_*i*_*≠m*_*j*_}, where em_ij_ is the edge between m_i_ and m_j_. This can be considered an extension of **Ep**.

Definition 6 Meter Graph **Gm**. An unweighted graph, **Gm**=(**M**, **Em**), is used to describe the topological structure of the meter nodes in the manufacturing system. **Gm** can be represented by an adjacency matrix **A** ∈ **R**^*N*×*N*^, which contains only elements of 0 and 1. The element in the *i*th row and the *j*th column is 1 if em_ij_ exists, and 0 otherwise. **Gm** is the extension of **Gp**, and **Em** is the extension of **Ep**. Constructing **Gm** is just the task to solve the **Q1**.

Definition 7 Energy-Consumption Time Series **X** ∈ **R**^*N*×*H*^. **X** = {*x*_*s*,*t*_|*m*_*s*_ ∈ **M**, *t* = 1,2, …,*H*}, where *x*_*s*,*t*_ is the energy-consumption value of the *s*th meter (m_s_) in the *t*th interval, H is the length of time series, **X** ∈ **R**^*N*×*H*^.

The definitions generalize the path of information dissemination. Spatially, energy consumption on the meter *m*_*s*_ and *m*_*u*_ (*m*_*u*_ denotes the *u*th meter and *u≠s*) can be connected by the path *x*_*s*,*t*_-*m*_*s*_*-p*_*i*_-*ep*_*ij*_-*p*_*j*_-*m*_*u*_*-x*_*u*,*t*_. Temporally, the energy consumption at different intervals can be connected by time sequence < *x*_*s*,*t*_, *x*_*s*,*t*+1_, *x*_*s*,*t*+2_, …>.

Short-term energy-consumption prediction for a manufacturing system can be expressed as follows:

(1) $$\hat{X}=f\left(A，,X\right)$$

where $\hat{X}$ ∈**R**^***N***×*F*^ is the predicted energy-consumption time series, *F* is the length of the predicted time series, and **f** is the function to be expressed by a learning model, which is the task to solve the **Q2**.

**Fig. 1:**
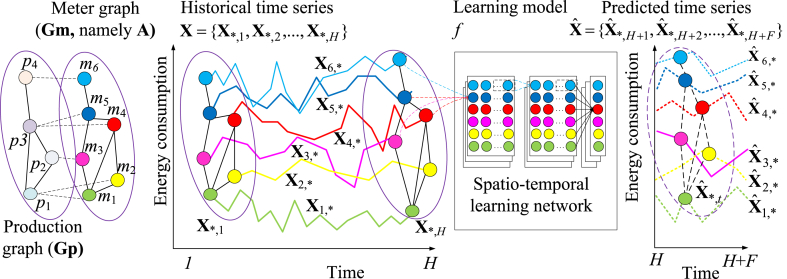
Conceptual structure of energy-consumption prediction for a manufacturing system.

Fig. 1 shows the conceptual structure of energy-consumption prediction in a manufacturing system. The input includes matrices **A** and **X**, **A** establishes the initial spatial connection between meters, and **X** represents the temporal states of the energy consumption of meters. Combined with **A**, **X** becomes an interconnected multinode time series instead of multiple independent time series. The output is the matrix $\hat{X}$, which is a multinode time series with a structure similar to **X**. **X** and $\hat{X}$ can be split into vectors from space (also meter) and time dimensions. Let X_*,*t*_ and ${\hat{X}}_{*,t}$ denote the energy-consumption profile in the *t*th interval (see the ellipses with the graph in Fig. 1), and **X**_*s*,*_ and ${\hat{X}}_{s,*}$ denote the energy-consumption time series on the meter *m*_*s*_ (see the curves in Fig. 1). Thereafter, **X** can be represented as $\left\{X_{*,1},X_{*,2},...,X_{*,H}\right\}$ and $\hat{X}$ can be represented as $\left\{{\hat{X}}_{*,H+1},{\hat{X}}_{*,H+2},...,{\hat{X}}_{*,H+F}\right\}$.

**A** is the pre-constructed graph for the subsequent GCN, and **f** is the learning network containing the GCN. Note that **Gp** is not the input of **f** but an intermediate of **A**, and the combination of **A** and **X** drives the spatiotemporal learning network. The construction for **A** (**Solution to Q1**) is described in Section 3, and the spatiotemporal learning network (**Solution to Q2**) is described in Section 4.

## 3 Sparse meter graph construction

**Fig. 2:**
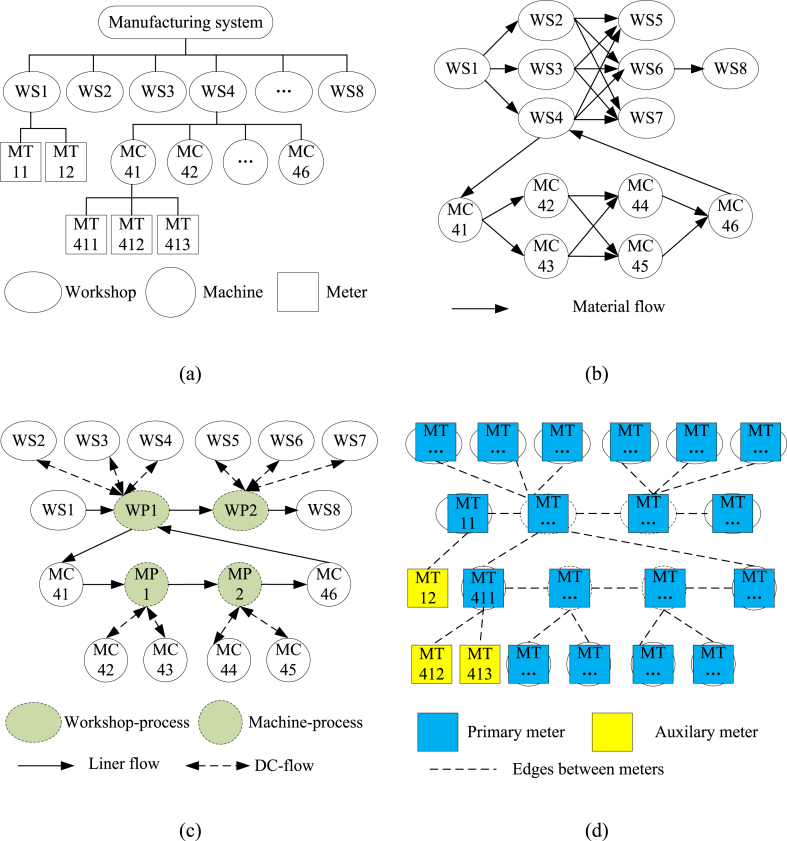
Example for the conceptual evolution of sparse meter graph. (a) Node hierarchy graph. (b) Original production graph. (c) Sparse production graph. (d) Sparse meter graph.

For energy consumption in manufacturing systems, the spatial relationship refers to the process path between machines. To ensure generality, a sparse meter graph was constructed for general production management and material flow rather than detailed technology knowledge. From a management perspective, a manufacturing system can be regarded as a hierarchical structure composed of plants, workshops, or individual machines [[54], [55], [56]], and energy consumption is transferred from the lower node to the upper node. From the perspective of the process, a manufacturing system can be regarded as a flow line, and the energy consumption is transferred from the upstream machines to the downstream machines [6]. Based on the above two ideas, the sparse meter graph undergoes a multistep evolution from the node hierarchy graph to the original production graph and then to a sparse production graph. An example of the multistep evolution of a sparse meter graph is shown in Fig. 2. The steps are described as follows.

Step 1 constructing node hierarchy graph.

A node hierarchy graph expresses hierarchical management and is described by node class, node object, and hierarchical relationships. The manufacturing system comprises numerous workshops, and each workshop comprises numerous machines. The production node classes and hierarchical relationships can be described as “*system*/*workshop*/*machine*”. The energy consumption of a production node is measured over numerous meters. Fig. 2(a) shows a node hierarchy graph instance in which the manufacturing system consists of eight workshops (identified as WS1 to WS8), workshop WS4 consists of six machines (identified as MC41, MC42, …, MC46), workshop WS1 is measured by 2 m (identified as MT11 and MT12), machine MC41 is measured by 3 m (identified as MT411, MT412, MC413), and the other machines and meters are omitted from the example.

Step 2 constructing original production graph.

An original production graph is derived from the original material flow and expressed the material flow at the workshop and machine levels. The material flow at the machine level starts from and ends with the upper workshop and thus builds a connection between the workshop and machine. In fact, the original production graph is locally dense owing to the parallel relationship among workshops (or machines). In a parallel relationship, numerous workshops (or machines) perform replaceable processes and share the source and destination of the material flow [57,58]. Fig. 2(b) shows an original production graph in which WS2, WS3, and WS4 are groups of parallel workshops, WS5, WS6, and WS7 are another group of parallel workshops, and the interlaced material flow can be seen between the two groups. Similar interlaced connections were also observed among machines MC42, MC43, MC44, and MC45. The interlaced connections tend to blur the spatial relationships between production nodes and introduce noise into the ensuing learning network; hence, the next task is to simplify and clarify the original production graph.

Step 3 constructing sparse production graph.

A sparse production graph is derived from the original production graph. This eliminates interlaced connections by defining the process node and refactoring the material flow. A process node is the upper node of a group of parallel workshops (or machines). The process node for parallel workshops is called a workshop-process node, and that for parallel machines is called a machine-process node. Inserting process nodes, the hierarchical structure of production node is extended from “*system/workshop/machine*” to “*system/workshop-process/workshop/machine-process/machine*”. Material flow is categorised into linear and distributing-collecting flow (D-C flow) flows. Linear flow indicates that the materials flow through each production node sequentially. The D-C flow means that materials are distributed from one upper production node to multiple lower production nodes and then returned to the upper production node. The interlaced material flow between two parallel groups can be converted into a composite of linear and D-C flows. Fig. 2(c) shows a sparse production graph converted from the original production graph. It can be seen that the interlaced connections in the original production graph are eliminated by adding process nodes (see WP1, WP2, MP1, and MP2) and refactoring material flow.

Step 4 constructing sparse meter graph.

A sparse meter graph is extended from sparse production and node hierarchy graphs. With a one-to-many relationship between the production node and meter, the production graph cannot be simply converted into a meter graph. Here, sibling meters are defined as meters that measure one production node in the node hierarchy graph. In addition to the connections in the sparse production graph, the sibling relationship must be described in the meter graph owing to the close correlation between the energy consumption of sibling meters.

However, an indistinguishable sibling relationship leads to dense connections not only between sibling meters but also between meters at two connected production nodes. Rahimifard et al. [6] categorised energy usage into primary and auxiliary. The former is the energy required to perform the process (e.g., melting a specific amount of metal during casting or removing a specific amount of material during machining), and the latter is the energy required by the auxiliary equipment for the process (e.g., generation of a vacuum for sand casting or pumping of coolant for machining). Accordingly, the sibling meters were split into one primary meter and the remaining auxiliary meters. A sparse production graph can be converted into a sparse meter graph by replacing the production node with its primary meter and connecting the auxiliary meter to its primary meter. Fig. 2(d) shows the sparse meter graph converted from the sparse production graph. Compared with the latter, the former adds auxiliary meters (see MT12, MT412, and MT413) and connections between the auxiliary meter and primary meter and converts the directed edges into undirected edges. The sparse meter graph is **Gm** and can be represented by the adjacency matrix **A**.

The energy consumption at the process nodes cannot be measured by a meter because they are virtual production nodes. To ensure the integrality of the energy consumption at the production node, a virtual meter is defined for a production node without a meter, and its energy-consumption time series is the sum of the lower nodes.

## 4 Symmetric spatiotemporal learning network

### 4.1 Symmetric network structure

**Fig. 3:**
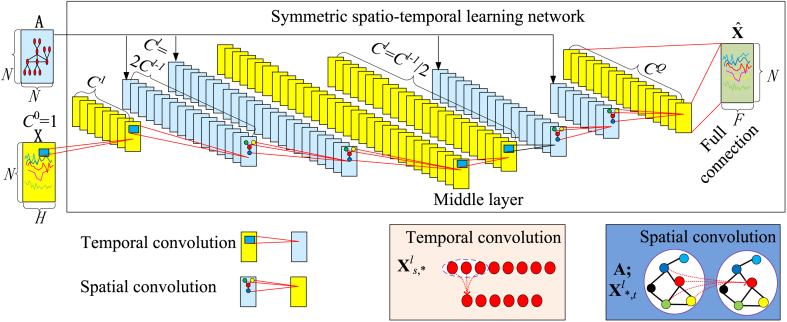
Structure of SSTLN.

The structure of the SSTLN is shown in Fig. 3. It comprised an even number of interleaved temporal and spatial convolution modules. Let **X**^*l*^ denote the input time series at the *l*th module, where l=0，,1,2,...,Q, *Q* is the number of modules and **X**^0^ = **X**. **X**^*l*^ can be split into **X**^*l*^_*s*,*_ and **X**^l^_*,*t*_ from the space and time dimensions. Temporal convolution is performed on **X**^*l*^_*s*,*_ and spatial convolution is performed on **X**^l^_*,*t*_ and **A** (mathematical expression of SMG). The details of each module are presented in the following subsections.

The symmetry of the network structure is explained as follows.

(1) The module layout is symmetrical. All convolution modules are arranged in “T-S-S-T- … -T-S-S-T” order in Fig. 3 (where T denotes temporal convolution module and S denotes spatial convolution module), and their reverse is the same arrangement. This design is expected to provide a trade-off between spatial and temporal feature extraction.
(2) The channel number assignment is symmetric. The number of channels is equivalent to the number of attributes of a node, and can be controlled by the number of convolution kernels in a module. For the *l*th module, *C*^*l−*1^ denotes the input channel number, and *C*^*l*^ denotes the output channel number. The number of channels is expanded in the first half of modules ($C^{l}=2C^{l-1}$) and symmetrically contracted in the second half of modules ($C^{l}=C^{l-1}/2$). But the modules at both ends are exceptional to adapt to the initial input and final output ($C^{0}=1$, $C^{1}$, and $c^{Q}$ are settable, and $c^{Q}$ denotes the output channel number of the last module). This design is expected to trade-off between detail extraction and noise suppression.
(3) The field of view of the convolution is symmetric. All temporal and spatial convolution kernels share the same kernel size. This design is expected to extract multigranularity features by stacking multilayer small convolution kernels.

### 4.2 Spatial convolution module

The GCN was chosen to construct the spatial convolution module because it has shown a strong capacity for capturing spatial features [40,41,42]. This module operates on **X**^l^_*,*t*_ and **A**. **A** is the mathematical expression for the SMG, and **X**^l^_*,*t*_ can be regarded as the input signal for the SMG. The hidden spatial features between neighbours are captured using graph convolution.

First, adjacency matrix **A** is converted into a normalised graph Laplacian matrix using the following formula [40]:

(2) $$L=I_{N}-D^{-\frac{1}{2}}AD^{-\frac{1}{2}}=U\Lambda U^{T}$$

where $L\in R^{N\times N}$ is the normalised graph Laplacian matrix, **I**_*N*_ is the identity matrix, $D\in R^{N\times N}$ is the diagonal degree matrix with $D_{ii}=\sum_{j}A_{ij}$, Λ is the diagonal matrix of the eigenvalues of **L**, and **U** is the matrix of the eigenvectors of **L**.

Then, graph convolution is operated, as follows [40]:

(3) $$X_{*,t}^{l+1}=\Theta^{l}*gX_{*,t}^{l}=\Theta^{l}\left(L\right)X_{*,t}^{l}=\Theta^{l}\left(U\Lambda U^{Τ}\right)X_{*,t}^{l}=U\Theta^{l}\left(\Lambda \right)U^{Τ}X_{*,t}^{l}$$

where “*g” is the graph convolution operator, $\Theta^{l}$ is the kernel that will multiply with the input signal matrix $X_{*，t}^{l}$. $\Theta^{l}$ is shared, and its *H* copies multiply with $\left(X_{*,t-H},X_{*,t-H+1},...,X_{*,t}\right)$.

To localise the filter and reduce the number of parameters, the kernel $\Theta^{l}$ can be restricted to a polynomial of Λ, as follows [40]:

(4) $$\Theta^{l}\left(\Lambda \right)=\sum_{k=0}^{K-1}\theta_{k}^{l}\Lambda^{k}$$

where $\theta^{l}\in R^{K}$ is a vector of polynomial coefficients and *K* is the kernel size of the graph convolution that determines the maximum radius of the convolution from the central nodes.

Generally, Chebyshev polynomial $T_{k}(\left(.\right)$ is used to approximate kernels as a truncated expansion of order *K*-1, as follows [40]:

(5) $$\Theta^{l}\left(\Lambda \right)\approx \sum_{k=0}^{K-1}\theta_{k}^{l}T_{k}\left(\tilde{\Lambda }\right)$$

where $\tilde{\Lambda }=2\Lambda /\lambda_{\max}-I_{N}$ is the rescaled of Λ, and $\lambda_{\max}$ denotes the largest eigenvalue of **L**.

The graph convolution can then be rewritten as follows [40]:

(6) $$\Theta^{l}\left(L\right)X_{*,t}^{l}\approx \sum_{k=0}^{K-1}\theta_{k}^{l}T_{k}\left(\tilde{L}\right)X_{*,t}^{l}$$

where $T_{k}\left(\tilde{L}\right)\in R^{N\times N}$ is the Chebyshev polynomial of order *k* evaluated at the scaled Laplacian $\tilde{L}=2L/\lambda_{\max}-I_{N}$ by recursively computing *K*-localised convolutions through a polynomial approximation. The graph convolution cost can be reduced from $Ο\left(N^{2}\right)$ to Ο(K×N).

Although the original input signal ($X_{*，,t}^{0}=X_{*,t}$) is a one-dimensional tensor, the channel number may be expanded or contracted during graph convolution; thus, the input signal $X_{*，,t}^{l}$ must be regarded as a multidimensional tensor. It is assumed that for the input signal $X_{*,t}^{l}\epsilon R^{N\times C^{l}}$ and output signals $X_{*,t}^{l+1}\epsilon R^{N\times C^{l+1}}$, the graph convolution can be generalised as follows [42]:

(7) $$X_{*,t}^{l+1,j}\sum_{i=1}^{C^{L}}\Theta_{i,j}^{l}\left(L\right)X_{*,t}^{l,j}$$

where $X_{*,t}^{l,i}\in R^{N}$, $X_{*,t}^{l+1,j}\in R^{N}$, $\Theta_{i,j}^{l}\in R^{K}$, $\Theta^{l}\epsilon R^{K\times C^{l}\times C^{l}+1}$.

### 4.3 Temporal convolution module

Although RNN-based algorithms are commonly used for capturing temporal features from time series, they suffer from complex gate mechanisms and time-consuming serial iterations. In addition, for a multinode energy-consumption series with complex temporal features, recursive feedback may introduce noise and slow convergence. Inspired by the traffic flow prediction method in Ref. [42], the GLU [59], which allows parallel matrix operation and multigranularity feature extraction, was chosen to construct the temporal convolution module. The module operates on **X**^*l*^_*s*,*_, which can be regarded as a time series on a single-meter node.

Similar to spatial convolution, **X**^*l*^_*s*,*_ must be regarded as a multidimensional tensor. Temporal convolution is performed as follows:

(8) $$X_{S,*}^{l+1}=\Gamma^{l}*\tau X_{S,*}^{l}=ReLU\left(\left(X_{S,*}^{l}*W^{l}+b^{l}\right)⊗\sigma \left(X_{S,*}^{l}*V^{l}+c^{l}\right)\right)$$

where “*τ” is the temporal convolution operator, $\Gamma^{l}\epsilon R^{K\times \left(C^{l+1}\right)\times 2\left(C^{l+1}+1\right)}$ is the kernel, *K* is the kernel size shared with spatial convolution, $W^{l}\epsilon R^{K\times C^{l}\times C^{l+1}}$, $V^{l}\epsilon R^{K\times C^{l}\times C^{l+1}}$, $b^{l}\epsilon R^{C^{l+1}}$, $c^{l}\epsilon R^{C^{l+1}}$ are half kernels and biases of $\Gamma^{l}$, $\Gamma^{l}=\left\{W^{l},b^{l}\right\}\cup \left\{V^{l},c^{l}\right\}$, ⊗ is the element-wise Hadamard product, σ is the sigmoid function, and ReLU is the rectified linear units function. $\Gamma^{l}$ is shared, and its *N* copies perform a temporal convolution with $X_{1,*}^{l},X_{2,*}^{l},...,X_{N,*}^{l}$.

The GLU contains two convolution operations, which are then combined by the ⊗ operation. The combined double convolution and sigmoid gate σ(.) contributed to the discovery of the compositional structure and dynamic variances in the time series.

### 4.4 Full connection module

The full connection module is the output layer of the learning network and maps $X^{Q}\in R^{\left(H-L\times \left(K-1\right)\right)\times N\times C^{Q}}$ to the prediction objective $\hat{X}\in R^{N\times F}$. It is independently operated on each meter node because the spatiotemporal features of each meter node are extracted to $X_{s,*}^{Q}$:

(9) $${\hat{X}}_{S,*}=X_{S,*}^{Q}*W^{Q}+b^{Q}$$

where $W^{Q}\in R^{\left(H-L\times \left(K-1\right)\right)\times C^{Q}}$ and $b^{Q}\in R^{F}$ are weights and biases.

In the forward process of the learning network, $\Theta^{l}$, $\Gamma^{l}$, $W^{Q}$, $b^{Q}$ are learning parameters, *Q*, *K*, $C^{1}$ and $C^{Q}$ are hyperparameters. The loss function is defined as follows:

(10) $$loss=\sqrt{\frac{1}{NF}\sum_{s=1}^{N}(\sum_{挺t=1}^{F}{\left(X_{s,t}-{\hat{X}}_{s,t}\right)}^{2}}+\lambda L_{\text{reg}}$$

where $X_{s,t}$ is the observed value of the *s*th meter node in the *t*th interval, ${\hat{X}}_{s,t}$ is the predicted value of the *s*th meter node in the *t*th interval, *L*_reg_ is the L2 regularisation term that helps avoid overfitting, and λ is a hyperparameter.

## 5 Application case

A large-scale aluminium-profile plant was chosen as the experimental case. This converts aluminium ingots into aluminium profiles applied in buildings or industrial products and consumes a large amount of energy, such as power, gas, diesel oil, water, and compressed air. The authors participated in the development of its energy management system, in which over 1000 energy meters were installed and data were collected minutely. The authors are authorised to read the technical documentation and access the energy-consumption dataset.

**Fig. 4:**
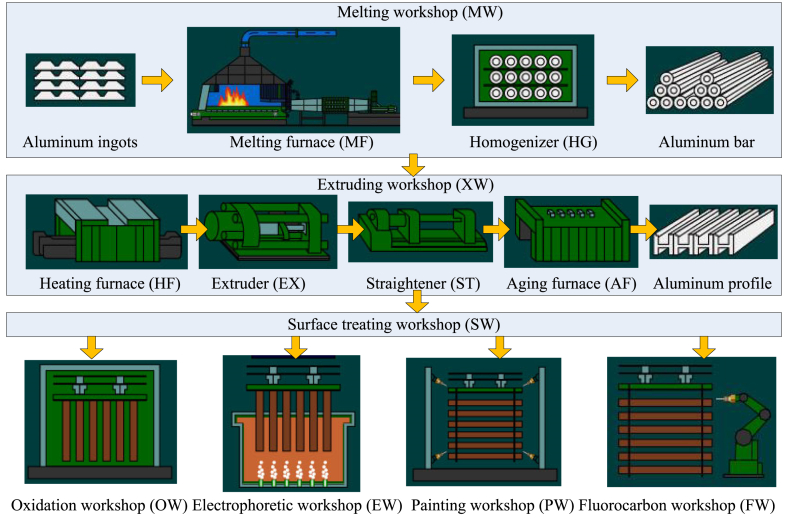
Process model of the studied aluminium profile plant.

The process model of the studied plant can be described by defining the workshop and machine process nodes, as shown in Fig. 4. At the workshop level, the process can be considered a linear flow consisting of melting, extrusion, and surface treatment. At the machine level, the processes in a melting workshop can be considered a linear flow consisting of a melting surface and homogeniser, and the processes in an extruding workshop can be considered a flow line consisting of a heating surface, extruder, straightener, and ageing furnace. The surface treatment workshop consisted of four parallel workshops: oxidation, electrophoresis, painting, and fluorocarbon workshops. Abbreviations for the workshops and machines are given in parentheses.

**Fig. 5:**
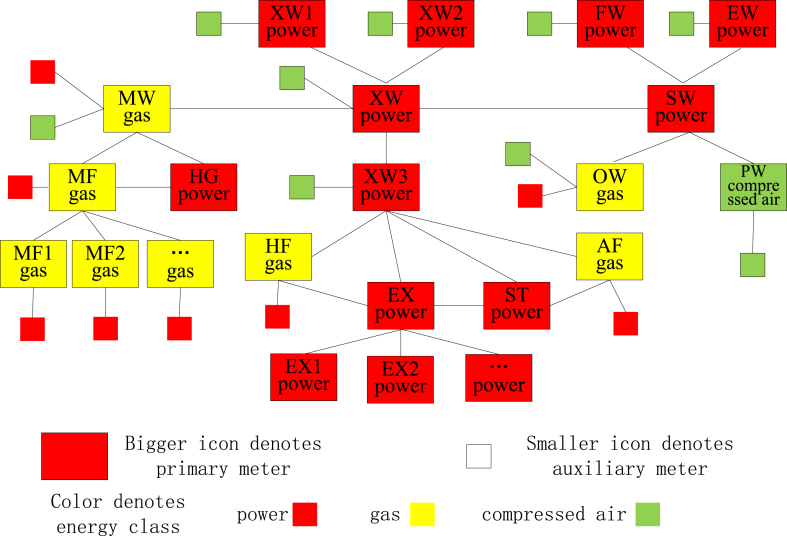
Part of the meter graph model of studied plant.

The primary and auxiliary energy consumption of the production nodes is listed in Table 1. Part of the SMG is shown in Fig. 5. The extruding workshop (XW) node is an abstraction of three parallel XWs (XW1, XW2, and XW3). Each XW node includes a linear flow consisting of HF, EX, ST, and AF, and each EX node includes parallel visible machines. Some nodes, for example, XW2 and XW3, are omitted. Each class of energy meter is represented as a colourful icon: power is red, gas is yellow, and compressed air is green. The larger icons with names indicate the primary energy meters, and the smaller icons without names indicate the auxiliary energy meters.

**Table 1:** Primary and auxiliary energy usage of production nodes.

Production nodes	Primary energy	Auxiliary energy
Melting workshop	Gas	Power, Compressed air
Melting furnace	Gas	Power
Homogeniser	Power	/
Extruding workshop	Power	Compressed air
Heating furnace	Gas	Power
Extruder	Power	/
Straightener	Power	/
Ageing furnace	Gas	Power
Surface treating workshop	Power	Compressed air
Oxidation workshop	Gas	Power, Compressed air
Electrophoretic workshop	Power	Compressed air
Painting workshop	Compressed air	Gas
Fluorocarbon workshop	Power	Compressed air

## 6 Experiment results and discussion

The presented method is evaluated to answer the following research questions.

● RQ1: From a prediction accuracy perspective, does the proposed SSTLN-SMG outperform state-of-the-art methods?
● RQ2: From a computation load perspective, can the proposed SSTLN-SMG meet the time requirements of short-term energy consumption prediction?
● RQ3: From a spatial pattern perspective, can the SMG help to extract the implicit relationships between node energy consumption?
● RQ4: From a predictive pattern perspective, can the symmetric structure and GLU in an SSTLN achieve better prediction accuracy than the empirical structure and RNN in multinode energy-consumption prediction?

### 6.1 Experimental settings

#### 6.1.1 Dataset

From the above application, 140 power meters, 24 gas meters, and 19 compressed air meters were selected for the meter set. A sparse meter graph (**A**) was constructed according to the method described in Section 3. The energy-consumption time series (**X**) over two months was periodically aggregated every 5, 15, 30, and 60 min. Each periodic dataset was regarded as an independent dataset and is briefly named the 5-min, 15-min, 30-min, and 60-min sets in the ensuing experiments. In the experiments, the input data are normalised to the interval [0, 1]. For each dataset, 70 % of the data were used as the training set, 15 % of the data was used as the validation set, and the remaining 15 % was used as the testing set.

#### 6.1.2 Learning network setting

The experiments were run on a workstation computer with a CPU Intel i7-11700 @2.5 GHz, GPU NVIDIA GeForce RTX 3080 Ti, RAM DDR 32G, and an operation system Ubuntu Linux. The presented SSTLN-SMG is developed using the Python language based on Tensorflow 2.0, and it reuses code provided by Ref. [42] on GitHub.

The length of the historical time series was set to 12 (*H* = 12) and the length of the predicted time series was set to 3 (*F* = 3). Empirically, the learning rate was set to 0.001, batch size to 64, training epoch to 200, and hyperparameters of the learning network were set to K=3, λ=0.001, and Q=16. $C^{1}$ and $C^{Q}$ were set to 16 and 128, respectively, in the dichotomous experiments.

#### 6.1.3 Baseline methods

The following baseline methods are compared with the presented SSTLN-SMG.

(1) **SVR** [19]: The kernel method based on support vectors performs well in time-series prediction, and SVR with a radial basis function kernel is used as the baseline method.
(2) **GRU** [23]: An RNN-based method for time-series prediction in which only temporal relationships are considered.
(3) **CGRUG** [30]: This is a hybrid network based on CNN and GRU and is designed for the multi-energy load prediction of energy systems. CNN modules were employed to extract spatial relationships, and GRU modules were employed to extract temporal relationships.
(4) **GCN** [40]: Several GCN modules are stacked on the pre-constructed SMG. It only considers spatial relationships.
(5) **GDN** [53]: This method is based on graph attention with dynamic graphs. The nodes of the graphs are represented by embedded vector learning from randomly initialised vectors, and the edges are established according to the similarity of nodes. Future values of each variate are predicted based on a graph attention function.
(6) **LPGNN** [51]: This is a hybrid network based on GCN and GLU with dynamic graphs. The GCN modules perform on dynamic graphs in which the nodes are represented by embedded vector learning from a multivariate time series, and low-pass and high-pass filters are added to alleviate the problem of node feature loss. The combination of GCN and GLU employs an empirical layout.
(7) **STSGCN** [45]: This is a hybrid network based on the GCN and GLU with pre-constructed graphs. The combination of GCN and GLU employs an empirical layout. In addition, it performs localized spatiotemporal graph fusion and embedding.

#### 6.1.4 Evaluation metric

Three metrics are used to predict the performance of the meter nodes and are defined as follows.

(1) Root-mean-square error (*RMSE*):

(11) $$RSME=\sqrt{\frac{1}{NF}\sum_{s=1}^{N}(\sum_{t=1}^{F}{\left(X_{s,t}-{\hat{X}}_{s,t}\right)}^{2}}$$

(2) Mean Absolute Error (*MAE*):

(12) $$MAE=\frac{1}{NF}\sum_{s=1}^{N}\sum_{t=1}^{F}\left|X_{s,t}-{\hat{X}}_{s,t}\right|$$

(3) Mean Absolute Percentage Error (*MAPE*):

(13) $$MAPE=\frac{100\%}{NF}\sum_{s=1}^{N}\sum_{t=1}^{F}\left|\frac{X_{s,t}-{\hat{X}}_{s,t}}{X_{s,t}}\right|$$

where the symbols have the same meanings as Eq. (10).

These three metrics are also used to predict the performance of a single node when *s* is certain and *N* is set to 1.

Specifically, the *RMSE* and *MAE* are used to measure the prediction error between the actual and predicted energy consumption, while *MAPE* is used to measure the relative error; the lower the value, the more stable the prediction result and the better the prediction effect.

### 6.2 Performance comparison (RQ1)

Each method was trained ten times, and the trained model with the minimum loss served as the final result. The prediction performances of the datasets for the application case are presented in Table 2. The best results for each dataset and metric are highlighted in bold, and the second-best results are underlined. ▴% denotes the improvement of SSTLN-SMG over the second-best method and is computed by the formula [(the second best-the best)/the second best × 100 %]. The prediction performance of a single node was evaluated and ranked using this method. Then, the nodes are counted using this method and ranked. The ranking counts of the presented SSTLN-SMG are shown in Table 3, where “124” in the upper left data cell means SSTLN-SMG achieves the best prediction performance for 124 nodes on the metric RMSE of a 5-min set. The observations were as follows.

● The SSTLN-SMG achieved the best performance on all metrics and the vast majority of single-node metrics. SSTLN-SMG achieved the best prediction performance for 67.76 %–91.80 % of the 183-m nodes and was the top three best for almost all meter nodes. The results verify that the presented SSTLN-SMG is effective on both the entire node set and a single node for multinode short-term energy-consumption prediction in the studied application case.

● Single-feature extraction methods (SVR, GRU, and GCN) underperformed spatiotemporal feature extraction methods (CGRUG, LPGNN, GDN, and SSTLN-SMG). The results verify the necessity of spatiotemporal feature extraction for short-term energy-consumption prediction in manufacturing systems. SVR emphasises the importance of modelling the relationship between input and output elements. GRU emphasises the importance of the time sequence and tends to capture temporal relationships from the time series. A GCN emphasises the importance of modelling spatial features and tends to capture hidden relationships among nodes from a single time profile with an initial spatial connection. Therefore, they failed to fully extract the spatiotemporal features hidden in the multivariate time series, resulting in poor prediction performance.
● The CNN-based CGRUG method underperformed GCN-based methods (LPGNN, GDN, and SSTLN-SMG). One possible reason is that the CNN establishes full connections between adjacent permutation nodes, which introduce noise into the training process. CNN was originally designed to extract local features from images. For multivariate time-series prediction, the presentation of local features depends on the permutation order of the variates, and it is difficult to arrange correlated variates in adjacent positions. A GCN is suitable for extracting global features from topological graphs and filtering noise during the training process through selective connections between nodes.
● The dynamic graph-based methods (LPGNN and GDN) underperformed the pre-constructed graph-based methods (the proposed SSTLN-SMG and STSGCN) on most of metrics. A possible reason for this is the false correlation between variates in the former. The connection of a dynamic graph is established based on the similarity between the time series of the variates. However, similarity cannot be used to measure the correlations between variates. As mentioned previously, two parallel machines may show similar energy-consumption fluctuations because they frequently share the same functionality and work rhythm; however, their energy consumption should be independent of each other because there is no material flow or ownership between them. Despite the elaborate learning network structure and learning strategy in LPGNN and GDN, SSTLN-SMG and STSGCN outperformed them by embedding simple prior knowledge into the pre-constructed graph.
● Observing the metrics of the spatiotemporal methods on the different periods, it can be concluded that GDN and STSGCN outperformed LPGNN on the 5-min set and 15-min set, and the results on the 60-min set were the opposite, and the results on the 30-min set were uncertain. Possible reasons are the change in correlation with the period and the difference in the methods of feature extraction. The time series on the shorter periods mainly reflect collaboration within a workshop and show stronger spatial correlation. As the period increases, the time series tends to reflect collaboration between workshops and show weaker spatial correlation. The GDN and STSGCN are biassed towards spatial feature extraction by performing graph attention function and fusing spatiotemporal graphs, respectively, and the LPGNN is biassed towards temporal feature extraction by node embedding from time series. The balanced strategy for spatiotemporal feature extraction may help the SSTLN-SMG outperform the LPGNN and GDN on all metrics.

**Table 2:** Prediction performances of methods on the datasets.

Dataset	Metrics	SVR	GRU	GCN	CGRUG	LPGNN	GDN	STSGCN	SSTLN-SMG	▴%
5-min	RMSE	14.69	6.87	30.33	5.31	13.58	7.50	5.22	**3.14**	39.92
	MAE	10.08	4.35	23.44	2.58	5.09	2.44	1.94	**1.61**	17.01
	MAPE	42.45	24.76	21.9	23.36	8.17	5.23	5.56	**3.74**	28.49
15-min	RMSE	43.63	29.36	59.48	23.17	21.02	19.19	9.54	**5.85**	38.68
	MAE	20.84	15.05	31.94	12.71	6.86	5.42	3.85	**1.86**	51.58
	MAPE	54.01	37.59	38.19	37.39	4.03	3.40	5.78	**1.99**	41.38
30-min	RMSE	126.04	51.73	88.52	34.08	22.37	27.91	14.87	**10.89**	26.77
	MAE	49.30	30.26	46.02	20.59	7.66	8.50	6.76	**4.04**	40.24
	MAPE	85.47	57.23	48.9	36.45	5.52	5.69	6.57	**4.29**	22.28
60-min	RMSE	236.47	110.67	104.14	65.35	25.12	35.77	28.08	**14.86**	40.84
	MAE	143.67	36.82	86.01	39.69	8.64	10.86	18.95	**8.10**	6.25
	MAPE	97.42	85.32	68.24	49.76	5.58	4.73	12.55	**3.71**	21.56

**Table 3:** Ranking counts of the SSTLN-SMG.

Dataset	Metrics	Ranking counts						
		1st	2nd	3rd	4th	5th	6th	7th
5-min	RMSE	124	48	11	0	0	0	0
	MAE	133	43	7	0	0	0	0
	MAPE	137	36	10	0	0	0	0
15-min	RMSE	168	14	0	0	1	0	0
	MAE	163	17	2	0	1	0	0
	MAPE	164	15	3	1	0	0	0
30-min	RMSE	151	30	2	0	0	0	0
	MAE	149	32	2	0	0	0	0
	MAPE	153	27	3	0	0	0	0
60-min	RMSE	163	14	4	2	0	0	0
	MAE	158	20	5	0	0	0	0
	MAPE	157	20	6	0	0	0	0

### 6.3 Computation load analysis (RQ2)

The time complexity of a learning network mainly depends on network structure. Obviously, the SVR, GRU, GCN and LPGNN have much lower time complexity than SSTLN-SMG since they consist of a single convolution operation. For the spatiotemporal networks, the main computational units are GCN and GLU, and the time complexity can be roughly evaluated by the number of spatial and temporal kernels. The SSTLN-SMG consists of 16 layers, and the number of kernel can be calculated by formula 16 × (2^7^+2^6^)+128 = 6240, according to symmetrical module layout. The STSGCN consists of 4 layers with 3 × 64 spatial kernels and 3 × 64 temporal kernels and the number of kernel can be calculated by formula 4 × 3 × 64 × 2 = 1536. However, the spatiotemporal graph is three times of the size of the spatial graph, and in terms of computation load the STSGCN is equivalent to having 1536 × 3 = 4608 kernels of the SSTLN-SMG. If taking spatiotemporal graph embedding into consideration, the computation load is not significantly different between the SSTLN-SMG and the STSGCN. In a similar way, the computation load of the LPGNN is equivalent to 3495 kernels and lower than the SSTLN-SMG. The CGRUG, the combination of CNN and GRU, is hard to compare with the SSTLN-SMG.

To verify the time consumption of the methods in application, the learning and predicting time are recorded in the experiments mentioned in Section 6.2, and the averages of each epoch on the 5-min dataset are shown in Table 4.

**Table 4:** Computation time spent in learning and predicting process (unit: seconds).

Time	SVR	GRU	GCN	CGRUG	LPGNN	GDN	STSGCN	SSTLN-SMG
Learning	28.0	23.7	26.1	42.2	18.3	2.7	72.3	**54.2**
Predicting	1.3	2.1	2.6	7.5	5.1	1.6	6.8	**5.1**

The experimental results are consistent with the analysis of time complexity. Observing the learning time, it can be seen that the learning time of the SSTLN-SMG is close to the STSGCN and the CGRUG, and is significantly longer than the other methods. The SSTLN-SMG takes about 3 h in a complete learning process with 200 epochs. Although the SSTLN-SMG does not show advantages over the baseline methods, it is acceptable since learning frequency is very low. Moreover, in actual applications, the learning time can be improved by upgrading hardware configuration.

Observing the predicting time, it can be seen that the predicting time of SSTLN-SMG is close to STSGCN, CGRUG and LPGNN, and is significantly longer than the other methods. The SSTLN-SMG takes about 5 s in a complete predicting process, and can meet the demand of minutely prediction.

### 6.4 Study of SMG (RQ3)

To prove the interpretability of SMG, the following deformed meter graphs were constructed to compare with SMG.

● **Dense meter graph (DMG)** is constructed based on the original production graph shown in Fig. 2(b). Missing the sparse production graph construction step, DMG is much denser than SMG.
● **Deformed graphs** are constructed by randomly adding or reducing 10 %, 20 %, 30 %, and 40 % of the edges on the SMG. They are called SMG^*z*^*,* where the superscript *z* denotes the proportion of changing edges; that is, SMG^*+10*^ and SMG^*−10*^ denote the graphs deformed by adding and reducing 10 % of the edges, respectively.

The comparison methods were constructed by replacing the SMG with the above graphs and were tested on each dataset to obtain a comparable set of performance metrics.

**Fig. 6:**
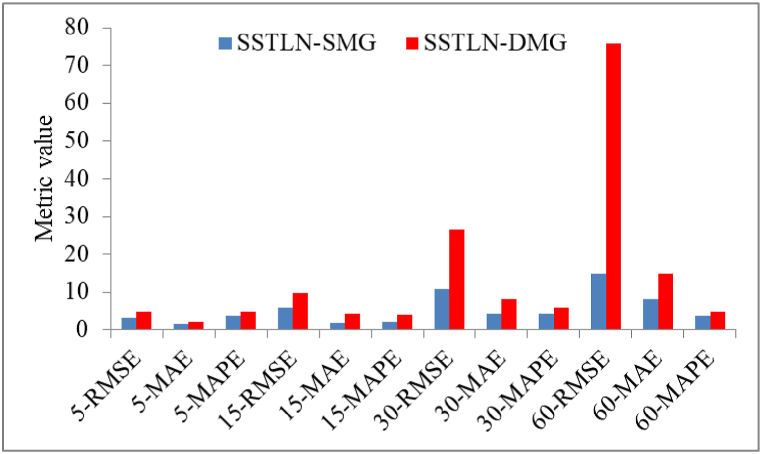
Prediction performance comparison between SSTLN-SMG and SSTLN-DMG.

**Fig. 7:**
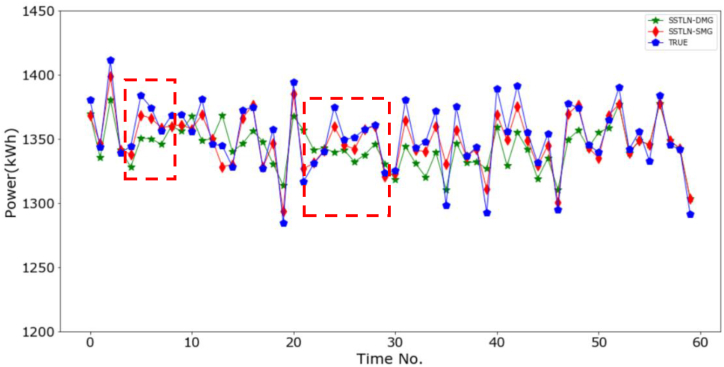
Continuous prediction curve of SSTLN-SMG and SSTLN-DMG for the 5-min set of a meter node.

**Impact of the graph sparsification steps**. Fig. 6 shows a performance comparison between the SSTLN-SMG and the SSTLN-DMG. The abscissa value is denoted as “dataset-metrics," e.g., "5-RMSE" means the RMSE on the 5-min set. It can be observed that the SMG outperforms the DMG on all metrics, and the DMG performs poorly on the 30-RMSE and 60-RMSE. A possible reason for this is the noise caused by the interlaced connections between meters in the DMG. The unnecessary connections in the DMG obscure the spatial relationship between energy consumption, introduce considerable noise into the deep learning process, and thus result in unstable and poor prediction performance. Fig. 7 shows the continuous prediction curves of the SSTLN-SMG and SSTLN-DMG on the 5-min set of a meter node. It can be found that the prediction curve of SSTLN-DMG shows rather great distortion in some time intervals (see the parts in the dotted boxes). The degree of the observed node is 3 in the SMG but 8 in the DMG, and unnecessary connections in the DMG may result in these considerable distortions.

**Fig. 8:**
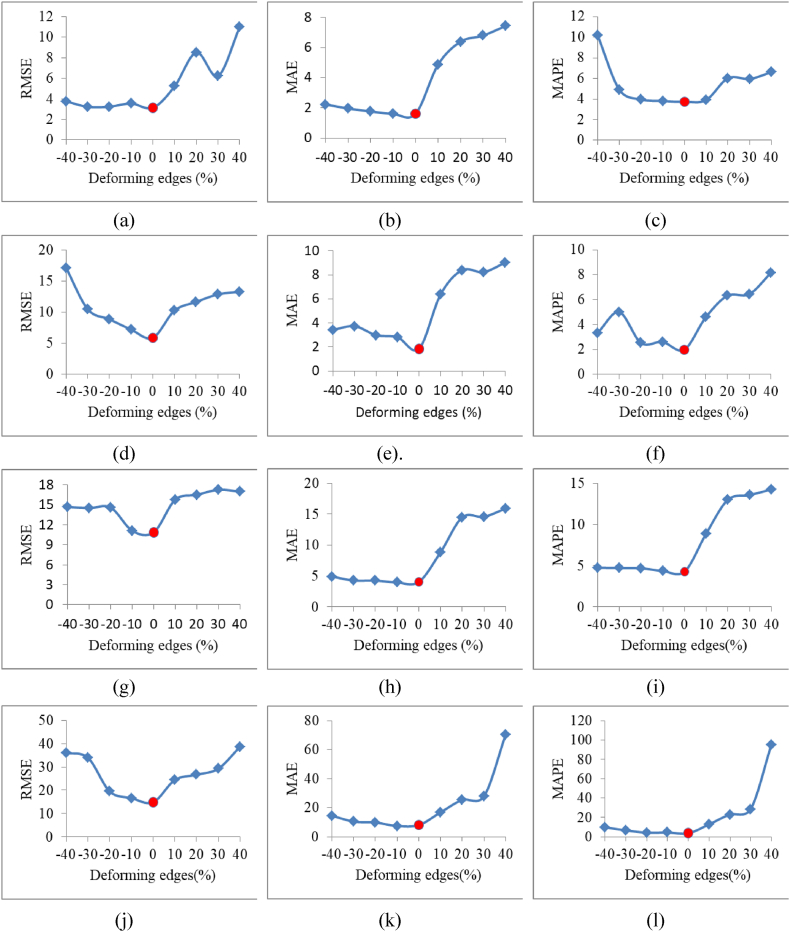
Prediction performance with changing edges. (a) RMSE on 5-min set. (b) MAE on 5-min set. (c) MAPE on 5-min set. (d) RMSE on 15-min set. (e) MAE on 15-min set. (f) MAPE on 15-min set. (g) RMSE on 30-min set. (h) MAE on 30-min set. (i) MAPE on 30-min set. (j) RMSE on 60-min set. (k) MAE on 60-min set. (l) MAPE on 60-min set.

**Necessity analysis of the connections in SMG**. Fig. 8 shows the prediction performance with changing edges, which contains 12 curves; four rows represent the four datasets, three columns represent the three performance metrics, and the points of the original SMG (at abscissa 0) are highlighted by red dots. The major conclusions from the curves are as follows: (1) The original SMG outperformed the deformed graphs for all metrics, validating the necessity of connections in the SMG. (2) In addition to the RMSE on the 5-min set (Fig. 8(a)) and MAPE on the 15-min set (Fig. 8(f)), the prediction performance degrades with the increase in deforming edges, which proves the reasonability of the connections in the SMG. (3) The prediction performance degrades more sharply with the addition of edges than with a reduction in edges. One possible reason for this is that the noise caused by the addition of edges has a more negative effect than the spatial feature loss caused by edge reduction.

### 6.5 Study of SSTLN (RQ4)

To prove the interpretability of the SSTLN, the following deformed learning networks were constructed for comparison with the SSTLN-SMG.

● **TST-SMG** replaces the symmetric module layout “T-S-S-T” with the layout “T-S-T”, sets the channel number assignment to {64,16,64} for each “T-S-T”, and sets the spatial kernel size to 1 and temporal kernel size to 3, which are recommended in Ref. [42].
● **STST-SMG** replaces the symmetric module layout “T-S-S-T” with the layout “S-T-S-T”, which is recommended in Ref. [2].
● **ZSTLN-SMG** replaces the channel number assignment with {16, 32, 48, 64, 80, 96,112,128}, which are linearly zoomed.
● **USTLN-SMG** replaces the channel number assignment with {128, 64, 32, 16, 16, 32, 64,128}, which is a U-shaped layout.
● **SSTGRU-SMG** replaces the GLU with the GRU recommended in Ref. [2] to extract temporal features.

Table 5 presents the prediction performances of the deformed learning networks for the datasets. It can be observed that the original SSTLN outperforms the deformed learning networks in all metrics, which validates the effectiveness of the presented SSTLN.

**Table 5:** Prediction performances of deformation learning networks on the datasets.

Dataset	Metrics	TST-SMG	STST-SMG	USTLN-SMG	ZSTLN-SMG	STGRU-SMG
5-min	RMSE	4.93	3.25	5.14	5.08	5.56
	MAE	2.11	1.69	1.85	1.96	2.99
	MAPE	5.24	5.01	3.90	8.00	7.18
15-min	RMSE	14.12	10.87	19.97	9.48	18.35
	MAE	3.85	4.51	5.00	4.25	9.36
	MAPE	4.58	4.15	2.56	2.76	24.41
30-min	RMSE	39.78	36.32	15.86	26.32	57.57
	MAE	15.98	4.79	6.95	6.36	22.74
	MAPE	23.26	4.78	5.49	9.67	30.58
60-min	RMSE	71.66	21.85	27.42	20.73	77.11
	MAE	26.59	9.26	10.98	33.54	43.71
	MAPE	6.58	5.56	5.07	12.49	41.20

**Fig. 9:**
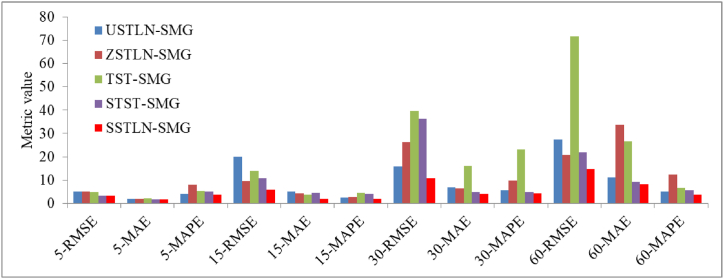
Prediction performance comparison among SSTLN-SMG and deformed learning networks.

**Fig. 10:**
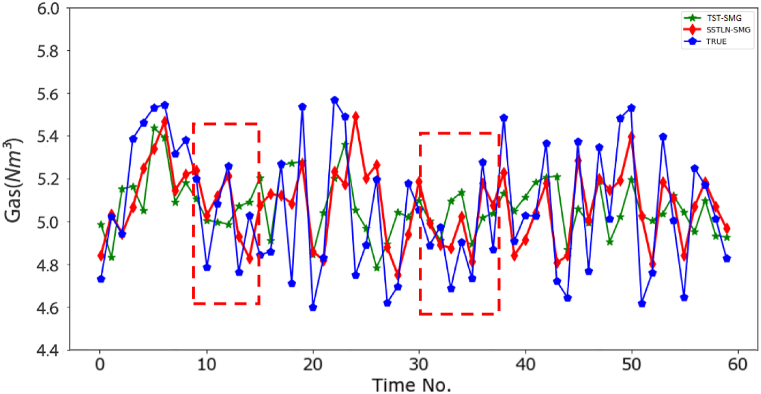
Continuous prediction curve of SSTLN-SMG and TST-SMG for the 5-min set of a meter node.

**Impact of the symmetric module layout**. Fig. 9 shows a comparison of the prediction performance of the SSTLN-SMG and deformed learning networks. The major conclusions from the bars are as follows: (1) The TST-SMG performed the poorest or second poorest on most of the metrics. A possible reason for this is that the TST-SMG modifies the module layout, channel number assignment, and kernel size. Moreover, the layout “T-S-T” and the channel number assignment {64, 16, 64} obviously lean towards temporal feature extraction. Fig. 10 shows the continuous prediction curves of the SSTLN-SMG and TST-SMG for the 5-min set of a meter node. It can be observed that the prediction curve of the TST-SMG shows a rather considerable distortion at certain time intervals (see the parts in the dotted boxes), which may be caused by the imbalance between temporal and spatial features, missing features, and unfiltered noises. (2) The USTLN-SMG and ZSTLN-SMG, with asymmetric channel number assignment, performed significantly worse than the SSTLN-SMG on most metrics, proving the advantage of symmetric channel number assignment in the SSTLN-SMG. (3) STST-SMG, with the module layout “S-T-S-T”, underperforms SSTLN-SMG on all metrics, which proves the advantage of the symmetric module layout in SSTLN-SMG.

**Fig. 11:**
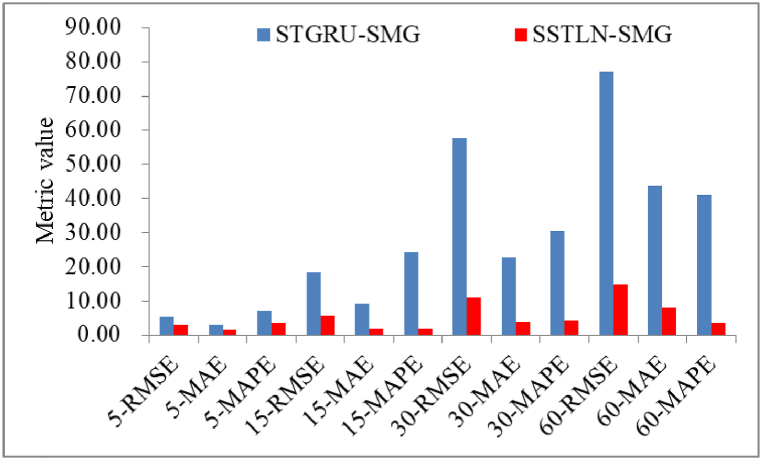
Prediction performance comparison between SSTLN-SMG and STGRU-SMG.

**Fig. 12:**
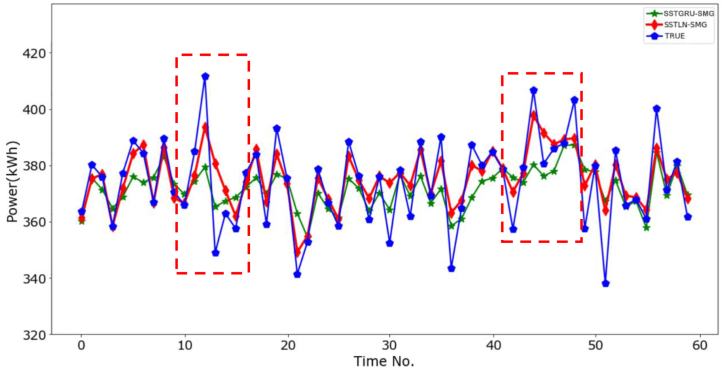
Continuous prediction curve of SSTLN-SMG and SSTGRU-SMG for the 5-min set of a meter node.

**Impact of the temporal convolution module**. Fig. 11 shows the prediction performance comparison between the SSTLN-SMG and STGRU-SMG. It can be observed that STGRU-SMG achieves significantly poorer performance than SSTLN-SMG on all metrics. These results prove that the GLU in the SSTLN-SMG is more effective for extracting temporal features than the GRU in the STGRU-SMG. Fig. 12 shows the continuous prediction curves of the two methods on the 5-min set of a meter node. The fluctuation trends of the two prediction curves are consistent with the true curve; however, the fluctuation amplitude error of the SSTLN-SMG is significantly less than that of the SSTGRU-SMG, particularly at the point with sharp fluctuations (see the parts in the dotted boxes). The GRU captures the long-term trend of a time series through serial output feedback. The GLU captures multigranularity features of the time series through multilayer and multichannel temporal convolution operations. Therefore, it can be concluded that the GRU is less sensitive to the extraction of local fluctuation features than the GLU. In the short-term energy-consumption time series in manufacturing systems, sharp local fluctuations exist owing to the dynamic production rhythm; thus, the GLU is more adaptable to this class of prediction tasks than the GRU.

### 6.6 Additional experiments on other datasets

To further validate prediction performance, the SSTLN-SMG was tested on the public datasets PEMSes, the district traffic flow used in the STSGCN. The STGCN [42] is also selected as a comparison method since it is the best baseline of the STSGCN.The comparative results are presented in Table 6.

**Table 6:** Prediction performances of methods on the PEMSes.

Dataset	MAE			RMSE			MAPE		
	STSGCN	STGCN	SSTLN-SMG	STSGCN	STGCN	SSTLN-SMG	STSGCN	STGCN	SSTLN-SMG
PEMS03	17.48	17.49	18.17	29.21	30.12	29.98	16.78	17.15	19.11
PEMS04	21.19	22.70	21.53	33.65	35.55	34.25	13.90	14.59	14.36
PEMS07	24.26	25.38	24.85	39.03	38.78	40.23	10.21	11.08	10.53
PEMS08	17.13	18.02	17.40	26.80	27.83	26.92	10.96	11.40	11.31

It can be found that the SSTLN-SMG slightly underperformed the STSGCN, but outperformed the STGCN on the datasets other than PEMS03. The results can be interpreted by the adaptability between dataset and network. The PEMSes are the datasets of district traffic flow with clear periodic patterns and road networks. The STSGCN enhances periodic pattern extraction by using spatiotemporal graph fusion. The STGCN weakens spatial feature extraction by using module layout “T-S-T” and channel number assignment {64, 16, 64}. The SSTLN-SMG balances spatial and temporal feature extraction by using symmetric network structure. Therefore, for the adaptability to the PEMSes, STSGCN > SSTLN-SMG > STGCN. Combing the previous experimental results, the SSTLN-SMG is able to adapt to various datasets, especially the datasets without clear periodic patterns and spatial relationships, like the short-term energy-consumption in manufacturing systems.

## 7 Conclusion

This paper presents a multinode, sequence-based, and learning-based method for short-term energy-consumption prediction in manufacturing systems that does not suffer from hard modelling work, limited data acquisition, and little prior knowledge. The main innovation lies in the construction of the sparse meter graph and the symmetric spatiotemporal learning network, which are designed to capture the complex spatiotemporal relationships among the production nodes. The SMG is constructed through evolving from material flow to meter connection. Compared with existing Petri net based methods, the presented method takes resource-centric modelling instead of process-centric modelling. It does not rely on process details and express explicit meter relationships in an intuitive way. The SSTLN employs a symmetrical module layout instead of an empirical layout, and aims to trade-off not only between spatial feature extraction and temporal feature extraction but also between details capture and noises suppression. The combination of SMG and SSTLN allows effective short-term energy-consumption prediction in manufacturing systems that can be used in multiple external applications (e.g., anomaly detection, real-time scheduling, and energy-saving control). Moreover, it is helpful for improving deep learning networks in other applications.

The method was validated using datasets from an aluminium profile plant that provided process documents and energy-consumption data. A sparse meter graph was constructed, and several experiments were performed. The results demonstrated that the proposed method outperformed other widely known methods, methods with deformed meter graphs, and methods with deformed learning networks for all metrics. In conclusion, for short-term energy-consumption prediction in manufacturing systems, a sparse meter graph can provide effective knowledge guidance for spatial feature extraction, and a SSTLN can effectively capture spatiotemporal relationships. In future work, we plan to establish an energy-consumption anomaly detection model based on this method.

## Data availability

Data will be made available on request.

## CRediT authorship contribution statement

**Jianhua Guo:** Writing – original draft, Supervision, Project administration, Methodology, Investigation, Funding acquisition, Formal analysis, Data curation, Conceptualization. **Mingdong Han:** Validation, Software, Methodology. **Chunlin Xu:** Writing – review & editing, Formal analysis. **Peng Liang:** Writing – review & editing. **Shaopeng Liu:** Writing – review & editing, Methodology. **Zhenghong Xiao:** Writing – review & editing. **Guozhi Zhan:** Writing – review & editing, Data curation. **Hao Yang:** Writing – review & editing.

## Declaration of competing interest

The authors declare that they have no known competing financial interests or personal relationships that could have appeared to influence the work reported in this paper.
